# 支气管肺泡灌洗在肺癌诊断方面的研究现状及展望

**DOI:** 10.3779/j.issn.1009-3419.2010.04.19

**Published:** 2010-04-20

**Authors:** 文涛 岳

**Affiliations:** 1 101149 北京，北京市结核病胸部肿瘤研究所暨北京胸科医院细胞生物学研究室 Department of Celluar & Molecular Biology, Beijing Tuberculosis and Thoracic Tumor Research Institute, Beijing Chest Hospital, Beijing 101149, China; 2 101149 北京，北京市结核病胸部肿瘤研究所暨北京胸科医院气管镜室 Department of Bronchoscope, Beijing Tuberculosis and Thoracic Tumor Research Institute, Beijing Chest Hospital, Beijing 101149, China

支气管肺癌是一种严重威胁人类健康和生命的疾病，其发病率有逐年增高的趋势，每年全世界约100万人死于肺癌。肺癌的早期诊断对提高患者5年生存率至关重要。传统胸部X线、CT影像学及痰液细胞学等检查在肺癌早期定性诊断方面具有一定的局限性。随着可弯曲支气管镜介入性检查手段在临床上被广泛采用，支气管镜检查逐渐成为肺癌早期诊断的重要手段。

常规支气镜检查包括经支气管钳夹活检（forceps biopsy, FB）、刷检（brush biopsy, BB）及经支气管针吸活检（transbronchial needle aspiration biopsy, TBNAB）等，钳夹活检、刷检可分别使74%、59%左右的肺癌明确诊断，但仍有部分肺癌，特别是周围型肺癌诊断较为困难^[1, 2]^。

支气管肺泡灌洗（bronchoalveolar lavage, BAL）是经支气管镜向局部支气管肺泡注入生理盐水，随即进行抽吸而获取支气管肺泡表面被覆液与灌入生理盐水混合液的一种方法。BAL可通过支气管肺泡灌洗液（bronchoalveolar lavage fluid, BALF）细胞学、肿瘤标志物等检测解决常规支气管镜未能见到的异常肺部病变的诊断，特别是周围性、原发性或继发性恶性肿瘤等，往往被称为“液相活检”^[3]^。

## 1 细胞学（cytology）

### 1.1 病理细胞学（pathocytology）

BAL时使用生理盐水灌注到更远端的细支气管肺泡，能大面积冲洗和收集肿瘤脱落细胞，进行BALF病理细胞学检查，可以提高周围性肺癌患者癌细胞检出率。为探讨支气管冲洗（bronchial washing, BW）在中心型肺癌及周围型肺癌诊断方面的价值，Lee等^[4, 5]^进行了研究，结果提示中心型可视肿瘤患者行BW，支气管冲洗液（bronchial washing fluid, BWF）肿瘤细胞发现率为55.8%-57.3%，FB未能明确诊断的6例患者却经BW发现肿瘤细胞而确诊；周围型不可视肿瘤患者行BW，肿瘤细胞发现率为20%。随后Poletti等^[6]^通过研究发现BALF标本腺癌检出率为80%。BALF检查被证明是诊断原发性、周围性肺部恶性肿瘤有价值的方法，并且肿瘤细胞学发现率与肺癌组织类型、大小、位置等有关^[5]^。

### 1.2 淋巴细胞及细胞因子（lymphocyte & cytokine）

肺部肿瘤的发生、发展与机体全身或局部免疫低下、肿瘤细胞发生免疫逃逸有关，与细胞因子水平关系密切。

为分析肺癌BALF中细胞成分概况与变化从而了解肺癌局部免疫反应，Domagała-Kulawik等^[7]^借助过氧化物免疫酶标记法应用CD3^+^等单克隆抗体对周围型肺癌患者及健康对照者进行研究，结果发现与健康对照者相比，肺癌患者BALF中巨噬细胞（60%）明显降低，淋巴细胞、中性粒细胞（24%、13%）明显增多，T淋巴细胞（86%）明显上升，CD4^+^/CD8^+^相对下降。随后，研究小组^[8]^又应用酶联免疫吸附测定（ELISA）法对38例原发性肺癌及23例健康志愿者的肿瘤生长因子-β1（tumor growth factor-β1, TGF-β1）进行了检测，研究发现肺癌患者BALF中TGF-β1浓度明显高于对照组，证实TGF-β1参与原发性肺癌的局部免疫反应，具有抑制宿主抗肿瘤免疫作用。

Katsoulis等^[9]^采用单克隆抗体方法对42例原发性肺癌患者及非恶性肿瘤患者的细胞因子进行检测，结果发现与血清中结果相比，肺癌患者BALF中肿瘤坏死因子-α（tumor necrosis factor-α, TNF-α）、白介素-1α（interleukin-1α, IL-1α）及白介素-6（interleukin-6, IL-6）增高（*P*=0.02），但对照组血及BALF中IL-6水平无明显差异（*P* < 0.05）。

Ohta等^[10]^应用ELISA法对41例原发性肺癌、7例非肺癌患者的血管内皮细胞生长因子（vascular endothial growth factor, VEGF）进行分析，发现肺癌患者BALF标本中VEGF浓度明显高于非肺癌患者（*P*=0.047），BALF标本中VEGF浓度均高于血浆，VEGF浓度不受性别、年龄、肿瘤部位及组织类型影响，而VEGF白蛋白标准化后的相对浓度即VEGF指数与肿瘤转移有关，Ⅰ期肺癌VEGF指数明显高于Ⅳ期（*P*=0.030 8）。检测BALF中的VEGF可了解气管内肿瘤血管生长信息，从而推断肿瘤发生发展。

### 1.3 凋亡能力（apoptotic capacity）

肺癌的发生、发展与细胞异常增殖和凋亡障碍有关。Zunic等^[11]^借助半定量细胞化学评分及指数法，应用末端脱氧核苷酸转移酶（TdT）介导的d-UTP缺口末端标记技术（TdT-mediated dUTP nick end labelling, TUNEL），对非小细胞肺癌（non small cell lung cancer, NSCLC）患者、抽烟及不抽烟的健康志愿者三组对象的肺组织中肺泡巨噬细胞产生或清除凋亡细胞的能力进行了评估，结果显示与不抽烟者（218.29±56.24）相比，抽烟者肺组织凋亡能力明显增强（289.55±50.77），与健康志愿者相比NSCLC患者肺组织凋亡能力明显减弱（150.30±40.61, *P* < 0.05）。

## 2 肿瘤标志物（tumor marker）

支气管肺泡上皮发生癌变时，可以产生或释放出某些肿瘤相关物质（异常基因及其产物）即肿瘤标志物于血清及支气管肺泡表面被覆液中。肿瘤标志物的研究与应用对肿瘤诊断及临床治疗开创了新领域。目前所应用的肿瘤标志物主要有胚胎类抗原、糖类抗原、细胞角蛋白、肿瘤相关的酶和激素、天然自身抗原以及某些癌基因等。

### 2.1 胚胎类抗原（carcinoembryonic antigen, CEA）

CEA被认为是胃肠道恶性肿癌的特殊抗原，因呼吸道上皮来源于胚胎内胚层，肺癌细胞也能直接产生。Golias等^[12]^应用免疫放射测定（IRMA）技术对50例肺癌、20例肺部良性病变及20例健康者进行CEA检测，结果发现肺癌患者BALF、血清中CEA水平明显高于其它两组（*P* < 0.05），肺部良性病变组吸烟与不吸烟个体间比较，抽烟者BALF中CEA增高且具统计学差异，而血清中无差异，并推论BALF中CEA水平受吸烟等其它因素影响，单独检测BALF中CEA来诊断肺癌具有一定局限性，应关注吸烟者癌变可能。

### 2.2 糖类抗原（carbohydrate antigen, CA）

CA是由一组糖基转移酶综合作用而产生的糖链抗原。CA125被认为是诊断卵巢癌敏感性和特异性较高的标志物，CA199主要作为直结肠和胰腺癌肿瘤标志物，CA153主要用于乳腺癌的监测和筛选及评价患者预后情况的指标。近年来，上述抗原的测定被更多地应用在肺癌诊断、治疗和预后观察上，取得了较好效果。

Dabrowska等^[13]^应用电化发光技术分别对NSCLC患者、矽肺患者及健康志愿者进行检测，结果显示NSCLC患者BALF中CA125水平明显高于其它两组，其敏感性、特异性分别为92%、80%。Solak等^[14]^以前瞻性研究方法选择51例肺癌、44例肺部良性疾病进行研究，发现肺癌患者BALF中CA153、CA199水平均明显高于良性对照组（*P* < 0.05），检测BALF中CA199的敏感性、特异性分别为29%、69%，CA153的敏感性、特异性分别为81.8%、68%。

### 2.3 多肽类抗原（tissue polypeptide antigen, TPA）

组织多肽抗原是从人体多种肿瘤组织中分离出的一种不含糖和脂的单链多肽抗原，在正常细胞中含量很少，但在上皮来源的增殖期肿瘤细胞中有很高的表达，对肺癌尤其是NSCLC的诊断、疗效观察及预后的判断具有很高价值。Prados等^[15]^研究了289例包括肺癌与非肺癌患者的BALF标本，并对细支气管灌洗液（bronchial lavage fluid, BLF）、肺泡灌洗液（alveolar lavage fluid, ALF）应用IRMA进行测定，结果显示原发性肺癌组BLF、ALF中TPA含量明显增高，吸烟肺癌患者ALF中TPA水平明显高于不吸烟者，但BLF中TPA水平无差别，TPA浓度与肺癌组织类型无关，推论TPA是一种很实用、有价值的肺癌标志物。

### 2.4 蛋白类抗原

细胞角蛋白19的片段（cytokeratin 19 fragments, CYFRA21-1）主要存在于上皮的细胞浆内，正常上皮细胞呈低水平表达，而源于上皮细胞的肿瘤如肺鳞癌等可过度表达此种蛋白，是NSCLC尤其是鳞癌的重要标志物。Dabrowska等^[13]^研究证明NSCLC患者BALF中CYFRA21-1水平明显高于健康对照组，但与矽肺组比较差别不大，CYFRA21-1在BALF标本中最佳甄别阈为3 ng/mL，并指出BALF中CYFRA21-1作为单一指标对肺癌诊断价值不大。

铁蛋白（ferritin, FT）是一种大分子蛋白复合物，各种肿瘤患者FT明显高于良性病变患者。Erbaycu等^[16]^对肺癌、肺结核及其它肺部良性疾病患者BALF中的FT水平进行了测定，发现三组患者BALF中FT水平无明显差异（*P*=0.584），中心型肺癌FT平均水平为73.56 ng/mL，周围性肺癌FT平均水平为31.54 ng/mL（*P*=0.087），肺癌组织类型、临床分期不影响BALF中的FT水平，BALF及血清FT无助于肺癌诊断。随后，Erbaycu等^[17]^通过研究进一步揭示血清FT（*P*=0.426）、BALF中FT（*P*=0.073）均与肺癌细胞类型无关，BALF中FT水平与肺癌患者生存期也无关。

### 2.5 肿瘤相关酶

神经元特异性烯醇酶（neurone specfic enolase, NSE）是糖酵解-烯醇化酶的同功酶，特异性定位于神经元和神经内分泌网状内皮系统细胞中，是神经源性肿瘤-神经母细胞瘤的肿瘤标志物。小细胞肺癌（small cell lung cancer, SCLC）是一种神经内分泌起源的肿瘤，可过量表达NSE。Karnak等^[18]^对35例肺癌、15例慢性阻塞性肺病（chronic obstructive pulmonary disease, COPD）及15例健康对照者进行前瞻性病例-对照研究，利用双抗体RIMA进行NSE检测，结果发现各组间血液、BALF标本中NSE水平无明显差异，肺癌组患者血液、BALF中NSE水平不因肺癌组织类型（小细胞与非小细胞）、临床分期（Ⅰ-Ⅱ期与Ⅲ-Ⅳ期）及有无吸烟史而有所差异，血液、BALF中NSE水平具有高度相关性，进而推断检测血液NSE水平足以诊断小细胞肺癌，尽管众所周知BALF中NSE为诊断NSCLC的重要参数。

### 2.6 鳞状细胞癌抗原（squamous cell carcinoma antigen, SCC）

SCC抗原是鳞状细胞癌特有抗原，其高表达多见于肺及食管鳞癌。Kim等^[19]^利用蛋白组学等技术研究了SCC表达情况，结果显示SCCA1、SCCA2在鳞癌患者气管、支气管上皮细胞中高表达，而在粘液细胞及正常气管、支气管上皮细胞中不表达。

### 2.7 肺癌相关基因

#### 2.7.1 端粒酶（telomerase）

人类端粒酶是由端粒酶RNA、催化亚单位（hTERT/Hest2）和端粒酶相关蛋白（TEP1）组成的核糖核蛋白复合体。在正常组织、肿瘤邻近组织和良性病变中端粒酶活性不表达或低活性表达，而在肺癌组织中呈现高表达。

Dikmen等^[20]^对22例肺癌、7例肺部良性病变患者进行研究，端粒酶活性检测的结果显示，肺癌组BALF中端粒酶活性阳性率为72.7%，肺良性疾病组阳性率14.3%（*P*=0.011）；端粒酶活性测定敏感性、特异性分别为72.7%、85.7%，阳性预测值为0.94，阴性预测值为0.50，诊断精确度为75.8%。BALF中端粒酶活性是具有很高敏感性的肺癌肿瘤标志物。

#### 2.7.2 基因甲基化（methylation）

近年来有关基因甲基化与肿瘤发生发展的关系日益受到人们的重视，成为肿瘤分子生物学研究的热点之一。基因甲基化主要发生在启动子CpG岛内CpG5'端胞嘧啶碱基上，CpG岛发生异常甲基化往往会改变基因的表达模式，导致抑癌基因的转录抑制或沉默。启动子异常甲基化是肿瘤发生的早期事件，*p53*、*p16*及*FHIT*（fragile histidine triad）等常发生甲基化^[21]^。

Kim等^[22]^为确定BALF中基因特异性甲基化对早期NSCLC的诊断价值，选择85例NSCLC患者及127例非肺癌患者，利用甲基化特异性PCR进行了BALF标本研究，结果显示68%NSCLC患者*p16*、*RASSF1A*（RAS association domain family protein 1）、*RARβ*（retinoic acid receptor β）及*H-cadherin*（H-钙粘蛋白）中至少1个基因甲基化，只有*FHIT*基因启动子甲基化与年龄和吸烟关系极为密切（*r*=0.36, *P*=0.03），并推论BALF中基因特异性甲基化为早期NSCLC诊断有价值的肿瘤标志物，存在于支气管上皮的与年龄有关的*FHIT*基因甲基化与烟草烟雾有关。

Topaloglu等^[23]^用逆转录聚合酶链反应（reverse transcriptase polymerase chain reaction, RT-PCR）检测BALF标本发现，非肺癌患者存在*RASSF1A* 、*CDH*1（E-cadherin）及*APC*（adenomatous polyposis coli promoter 1 A）的低水平异常甲基化，肺癌患者68%异常甲基化，并指出多数肺癌BALF标本中均能检测出甲基化，需要在肺癌早期诊断中进一步评价。

de Fraipont等^[24]^对49例NSCLC术后患者及77例肺癌高危人群的支气管灌洗液（bronchial lavage, BL）标本的*p16*、*DAP*K（death-associated protein kinase）、*MGMT*（O6-甲基鸟嘌呤-DNA甲基转移酶）、*FHIT*和*APC*基因甲基化进行了研究，结果显示49%的BL呈现阳性结果，周围型肺癌38%为阳性，中心型肺癌73%为阳性，1/3患者早期*FHIT*即呈阳性，*APC*甲基化较晚出现，且与*MGMT*一样在BALF中很少表达，*p16*甲基化率为17%，*DAPK*甲基化率为15%，14例肺癌患者术后恢复正常水平，3例复发中心型NSCLC患者BALF呈现阳性。结论提示BL标本基因甲基化有助于中心型肺癌诊断，但周围型肺癌CT扫描具有优势。

Schmiemann等^[25]^进一步进行了原发肺癌与复发肺癌、中心型肺癌与周围型肺癌支气管抽吸液*APC*、*p16INK4a*（cyclin-dependent kinase inhibitor-2A）和*RASSF1A*基因甲基化的对比研究，利用甲基化特异性实时定量PCR（quantitative methylation-specific real-time PCR, QMSP）法进行了联合检测，结果显示63%中心型肺癌、44%周围型肺癌、71%复发性肺癌基因甲基化检测呈现阳性反应，35%细胞学、组织学未能诊断明确的周围型肺癌通过甲基化阳性而确诊，非肺癌患者只有1例呈现假阳性反应，提示QMSP法基因甲基化检测有助于周围型肺癌的诊断。

## 3 BAL在诊断肺癌方面的局限性

虽然BALF标本离心上清液利用ELISA、IRMA及酶免疫等方法，沉淀物应用细胞分析、PCR扩增及TRAP等技术在检测指标上取得了初步可喜结果，但现行BALF尚存在一定局限性：①BALF回收量具可变性且BAL过程可以改变BALF中成份的浓度；②BALF标本单一肿瘤标志物等检测指标的敏感性、特异性较低；③除基因methylation等为肺癌全和无现象外，其余大多数肿瘤标志物在肺癌以外其它肺部疾病中也可表达；④BAL时所收集的BALF中肿瘤细胞含量较少，限定了通过基因组学等各种肿瘤标志物进行肺癌诊断和鉴别诊断的难度等。

## 4 展望

上述局限性为BALF在肺癌早期诊断方面提出了挑战。随着细胞生物学、免疫学及分子生物学等学科的迅猛发展、各种先进检测技术手段的逐渐采用^[26]^及BAL技术的不断规范与完善^[27, 28]^，相信BALF在肺癌早期诊断方面存在的不足将不断被解决、完善，其应用前景较为广阔。
